# Combination therapy with vancomycin-loaded calcium sulfate and vancomycin-loaded PMMA in the treatment of chronic osteomyelitis

**DOI:** 10.1186/s12891-016-1352-9

**Published:** 2016-12-22

**Authors:** Shanchao Luo, Tongmeng Jiang, Yingnian Yang, Xiaoping Yang, Jinmin Zhao

**Affiliations:** 1 Department of Orthopaedics Trauma and Hand Surgery, The First Affiliated Hospital of Guangxi Medical University, 530021 Nanning, Guangxi China; 2 Yulin Orthopedics Hospital of Chinese and Western Medicine, 537000 Yulin, Guangxi China; 3 Guangxi Engineering Center in Biomedical Materials for Tissue and Organ Regeneration, Guangxi Medical University, 530021 Nanning, Guangxi China; 4 Collaborative Innovation Center of Guangxi Biological Medicine, Guangxi Medical University, 530021 Nanning, Guangxi China; 5 Guangxi Key Laboratory of Regenerative Medicine, Guangxi Medical University, 530021 Nanning, Guangxi China

**Keywords:** Osteomyelitis, Debridement, Antibiotic, Calcium Sulfate, PMMA, Level of Evidence Level III, Retrospective trial

## Abstract

**Background:**

Chronic post-traumatic and postoperative osteomyelitis is a refractory disease which results in significant morbidity and mortality. The effect of combination therapy with vancomycin-loaded calcium sulfate and vancomycin-loaded polymethyl methacrylate (PMMA) was unknown.

**Methods:**

Fifty-one patients suffering from chronic post-traumatic or postoperative osteomyelitis of the lower extremities were included in the retrospective investigation. The patients were assigned to the study group of the combination therapy with antibiotic-loaded calcium sulfate and antibiotic-loaded PMMA or the control group of the antibiotic-loaded PMMA. Hematological parameters, eradication of infection, rate of infection recurrence and reoperation rate were evaluated during the follow-up.

**Results:**

The cases were followed up for an average of 24 months (range, 15–48 months) after the first-stage surgical operation. In the study group, all the patients revealed complete calcium sulfate resorption at an average of 6 weeks (range, 30–60 days). In the study group, infection was primarily eradicated in 92.31% (24 of 26) of patients and re-operation rate of 7.69% (2 of 26) after the first-stage surgery. Two patients underwent further surgical operation in the study group. One case achieved infection eradication in the recurrent two cases, with a secondary infection eradication rate of 96.15% (25 of 26). There was no persistent infection in the study group. In the control group, infection was eradicated in 64.00% (16 of 25) of patients and re-operation rate was 36.00% (9 of 25) after the first-stage surgery. Nine patients in the control group underwent further surgical operation. Two case achieved infection eradication in these cases who suffered from persistent or recurrent infection, with a secondary infection eradication rate of 72.00% (18 of 25). There was more re-operation rate in the control group (PMMA group, 9 vs combination therapy group, 2; *P* = 0.034).

**Conclusion:**

The combination therapy with vancomycin-loaded calcium sulfate and vancomycin-loaded PMMA possibly achieved more effective control of infection in the treatment of osteomyelitis through synergistic effect. The immediate structural stabilization and higher concentration of antibiotic at the local site of infection may be achieved through the combination of biodegradable and non-biodegradable devices in the treatment of chronic post-traumatic and postoperative osteomyelitis.

The study was retrospectively registered at 11/16/2016 (TRN: NCT02968693).

## Background

Chronic post-traumatic and postoperative osteomyelitis is a devastating complication of Orthopaedic trauma and reconstructive Orthopaedics, and is one kind of osteomyelitis with increasing number [1]. It is specially difficult to treat because of its refractory nature and complexity of diagnosis and treatment. Currently, proper treatment for chronic post-traumatic and postoperative osteomyelitis requires surgical debridement, elimination of dead space with local antibiotic delivery devices, which are obtained by implantation of antibiotic-loaded material, and systemic antibiotic therapy. Although systemic antibiotic therapy is an important part of the standard strategy, its effect can be impaired due to the poor penetration and poor blood supply at the local site of infection [2]. High-dose and long-term antibiotic therapy is related to severe systemic adverse effects, and long-term usage of antibiotics can promote the growth of bacterial drug resistance [3]. Local antibiotic therapy is one of the most described strategies for these drawbacks. It can reduce the risk of adverse effects caused by systemic antibiotic. Antibiotic-loaded bone cavity fillers which are consisted of degradable or non-degradable materials can be used for local antibiotic delivery devices. This not only fills up the bone cavity after surgical debridement, but also can reduce antibiotic concentration in serum and offer high concentration of antibiotic at the local site of infections [4–8]. Antibiotic-loaded PMMA has been the gold standard vehicle for local antibiotic delivery in the treatment of chronic osteomyelitis, because the antibiotic delivery system can offer the advantage of local release of high antibiotic level at the site of infection, and simultaneously enable the dead space originating from surgical debridement.

However, while PMMA was wildly employed, there are some disadvantages to be found. One of these disadvantages is the fact that PMMA can offer a substrate for bacterial colonization, especially when the release of antibiotic loaded in PMMA declines over time [9] and finally becomes ineffective. Another disadvantage is that, it is difficult to achieve and maintain the required concentration of antibiotic for the desired duration of time, because the release of antibiotics loaded in PMMA will become invalid over time and the antibiotics loaded in PMMA cannot be released completely. A high release of antibiotic from PMMA showed in the initial stage, and subsequently comes to an ineffective and constant rate of elution [10]. Additionally, the two-stage revision is needed to performed for removal the PMMA because of its non-degradation. These disadvantages of non-biodegradable PMMA have led to the pursuit for absorbable antibiotic delivery alternatives. Calcium sulfate loaded with antibiotic is one of the most extensively degradable carriers employed in the treatment of chronic osteomyelitis. It can achieve obliteration of the dead space resulting from surgical debridement, and release their antibiotic completely after their entire degradation, lacking of substratum for bacterial colonization. In addition, it is not required to be removed once again, owing to their inherently biodegradable nature. A recent study revealed that the calcium sulfate particles loaded with antibiotic had equal efficacy in the treatment of chronic osteomyelitis, comparing with PMMA loaded with antibiotic [11]. Given inefficient release kinetics of PMMA, calcium sulfate pellets loaded with antibiotic can perform a zero-order release kinetics which is not beneficial to the growth of antibiotic-resistant bacterial strains, owing to leaving no prolonged, low-level tail-release of antibiotic. Moreover, as one kind of surface eroding materials, calcium sulfate primarily releases antibiotic in their outer rim through degradation layer by layer, therefore the release rates of antibiotic loaded in calcium sulfate are relatively constant and sustained at a therapeutic level during a long-term period of time. However, antibiotic delivery devices made of calcium sulfate have intrinsic disadvantages. First, calcium sulfate cannot provide structural support for the stabilization of the bony structure, especially in the gradual degradation of calcium, the mechanical support further weakening. However, the mechanical stabilization of the bony structure is considerably significant in the treatment of chronic osteomyelitis [12]. Antibiotic-loaded PMMA spacers can support the bony structure with immediate stabilization or are advantageous to the maintaining of the skeletal stabilization. Antibiotic-loaded PMMA spacers can maintain the length of the bony structure and soft-tissue at the local site of infection, owing to their non-degradation. It may be advantageous to the secondary surgical revision.

### Hypothesis

However, to our knowledge, there haven’t been any studies using combination therapy with antibiotic-loaded calcium sulfate pellets and antibiotic-loaded PMMA spacers for the treatment of chronic osteomyelitis. We hypothesized that combination therapy with antibiotic-loaded calcium sulfate pellets and antibiotic-loaded PMMA spacers may generate a synergistic effect in the treatment of chronic osteomyelitis through combined application of the respective advantages of calcium sulfate pellets and PMMA spacers. The primary objective of this retrospective study (Level of Evidence Level III) was to compare the efficiency and safety of combination therapy with vancomycin-loaded calcium sulfate pellets and vancomycin-loaded PMMA spacers with vancomycin-loaded PMMA spacers in the treatment of chronic post-traumatic and postoperative osteomyelitis.

## Methods

### Inclusion/exclusion criteria

The retrospective study included patients with chronic post-traumatic or postoperative osteomyelitis and excluded patients suffering from hematogenous osteomyelitis or acute post-traumatic or postoperative osteomyelitis. Between April 2011 and April 2015, fifty-one patients requiring surgical treatment for chronic post-traumatic/postoperative osteomyelitis of the lower extremities were enrolled in a retrospective and control study in our hospital. The study was approved by the hospital’s ethical review committee. In this retrospective study, all the patients possessed clinical and radiographic evidence of chronic osteomyelitis for more than 3 months.

### Preparation of calcium sulfate pellets and PMMA spacer

To obtain a paste suitable for pellets, the following steps should be used: 1. Empty 10 cc stimulan® calcium sulfate powder (Stimulan; Biocomposites Ltd; United Kingdom) into a sterile mixing bowl. 2. The calcium sulfate powder was mixed with 2,000 mg of vancomycin powder. 3. Add approximately 5 ml mixing solution into the above mixture. Mix thoroughly until a smooth paste is formed (approximately 30 s). 4. The resultant paste is uniformly smooth into the mould provided to form pellets with diameters of 4.8 mm and height of 3.3 mm. 5. Allow paste to cure undisturbed for at least 15 min after mixing. Flex mould to release pellets.

One sachet of 40 g PALACOSR® + G power containing 33.6 g PMMA and with the addition of 0.5 g gentamicin sulphate (Heraeus Medical GmbH, Germany) was mixed with 4,000 mg of vancomycin powder in a sterile bowl. The liquid provided was poured into the resultant mixture above. Then, the mixture was stirred carefully for 30 s. If the dough-like mass no longer sticked to the rubber gloves, it can be progressed. If the required consistency was obtained, the cement can be applied to the bony defect until it hardened completely.

### Patients group assigned and demographics

The patients included in this retrospective study were divided into two groups according to antibiotic delivery devices employed, the vancomycin-loaded PMMA spacers group (with the addition of gentamicin sulphate) and the combination therapy with vancomycin-loaded calcium sulfate and vancomycin-loaded PMMA spacers group (with the addition of gentamicin sulphate). The vancomycin-loaded PMMA spacers with the addition of gentamicin sulphate group included 25 cases (14 male, 11 female) with an average age of 42.32 years (range, 17–68 years). Four of the cases had a history of diabetes, six were active smokers, three had osteoporosis, five was obese and no one had a history of substance abuse. The combination therapy with vancomycin-loaded calcium sulfate and vancomycin-loaded PMMA spacers group (with the addition of gentamicin sulphate) comprised of 26 patients (14 male, 12 female) with an average age of 43.84 years (range, 15–64 years). Five of the patients had a history of diabetes, six were active smokers, four had osteoporosis, four were obese and no one had a history of substance abuse.

### Patient’s classification

The PMMA spacers group included 25 patients who suffered from chronic post-traumatic or postoperation osteomyelitis in origin, and all the patients had undergone a mean of 3.2 previous surgeries on the infected site (range, 1–8). Internal fixation has been removed in 15 patients. There were ten patients with infected nonunion. Thirteen patients with closed fracture encountered post-traumatic or postoperative infection, and 12 patients with open fracture encountered post-traumatic or postoperative infection. The infection foci located in femur 5, tibia 17, calcaneus 3. Bone defect/void size after surgical debridement ranged from 11.70 to 168.00 cm^3^ with a average of 63.11 cm^3^.

In the combination therapy with calcium sulfate and PMMA spacers group, 26 patients who suffered from chronic post-traumatic or postoperation osteomyelitis in origin, and patients had undergone a mean of 3.4 previous surgeries on the infected site (range, 1–9). Internal fixation has been removed in 16 patients. There were 10 patients with infected nonunion. 12 patients with closed fracture encountered post-traumatic or postoperative infection, and 14 patients with open fracture encountered post-traumatic or postoperative infection. The infection foci located in femur 4, tibia 19, calcaneus 3. Bone defect/void size after surgical debridement ranged from 10.30 to 163.00 cm^3^ with a average of 62.73 cm^3^.

The Cierny-Mader classification system was used to classify patients’ infections [13]. The PMMA spacers group comprised of 10 patients with Stage I medullary osteomyelitis, 8 patients with Stage III localized osteomyelitis, and 7 patients with Stage IV diffuse osteomyelitis. In the combination therapy with calcium sulfate and PMMA spacers group, 11 patients had Stage I medullary osteomyelitis, 9 had Stage III localized osteomyelitis, and 6 had Stage IV diffuse osteomyelitis.

### Surgical treatment

The lead author performed all procedures. All the patients underwent two stage surgical treatments. In the first-stage treatment, the surgical treatment of infection was performed under a standard protocol, with multiple consecutive operative steps: Removal of the internal fixation material, debridement of infected soft tissue and necrotic bone tissue (with soft tissue and bone tissue for bacterial culture and histopathological inspection), resection of sclerotic bone within the infected site with high-speed burr, washing with wound pulse irrigation system, obliteration of dead space with PMMA spacer or combination PMMA spacer with calcium sulfate pellets, implantation of drainage tube, wound closure, refixation. Except for the material implanted the bone space, the principles and sequence of surgical operation for the two groups above were identical.

Some of the patients with infected nonunion before surgery or having a high risk of fracture after surgery were treated with external fixation material. Skeletal stabilization was performed appropriately in both groups: 20 patients with infected nonunion received external fixation (15 unilateral external fixation and 5 Ilizarov frames), and 15 patients with a high risk of fracture after surgery received external fixation (10 unilateral external fixation and 5 plaster fixation). The patients without infected nonunion before surgery or having no a high risk of fracture after surgery were not treated with external fixation material. The drainage tubes inserted in each patient were retained for 24 h postoperatively.

### Implantation of bone filler

**Fig. 1:**
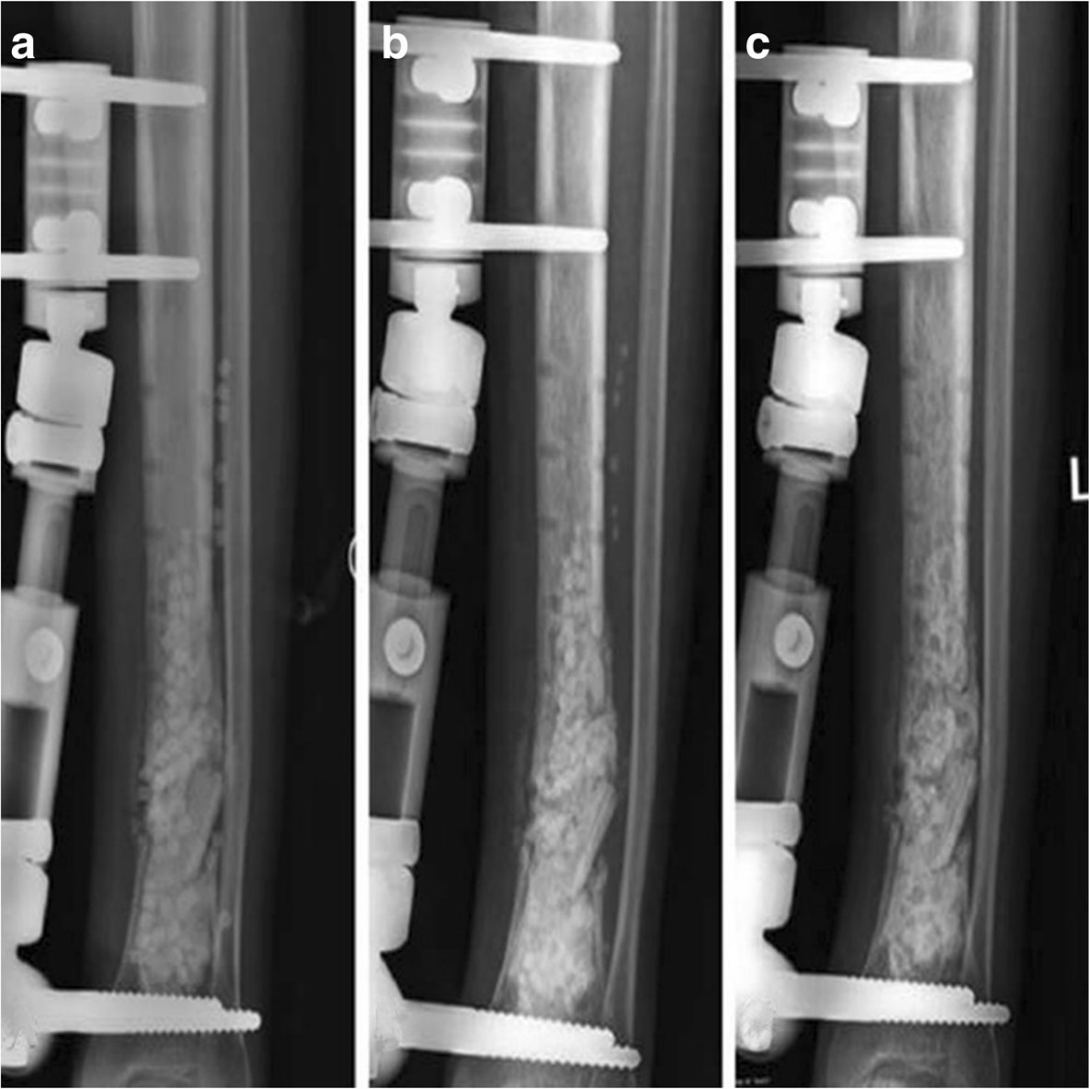
**a**-**c** Postoperative radiographs showed that the combination of calcium sulfate pellets and PMMA spacer was filled in the bone cavity after surgical treatment, and the calcium sulfate pellets were absorbed at an average of 6 weeks (range, 30–60 days)

**Fig. 2:**
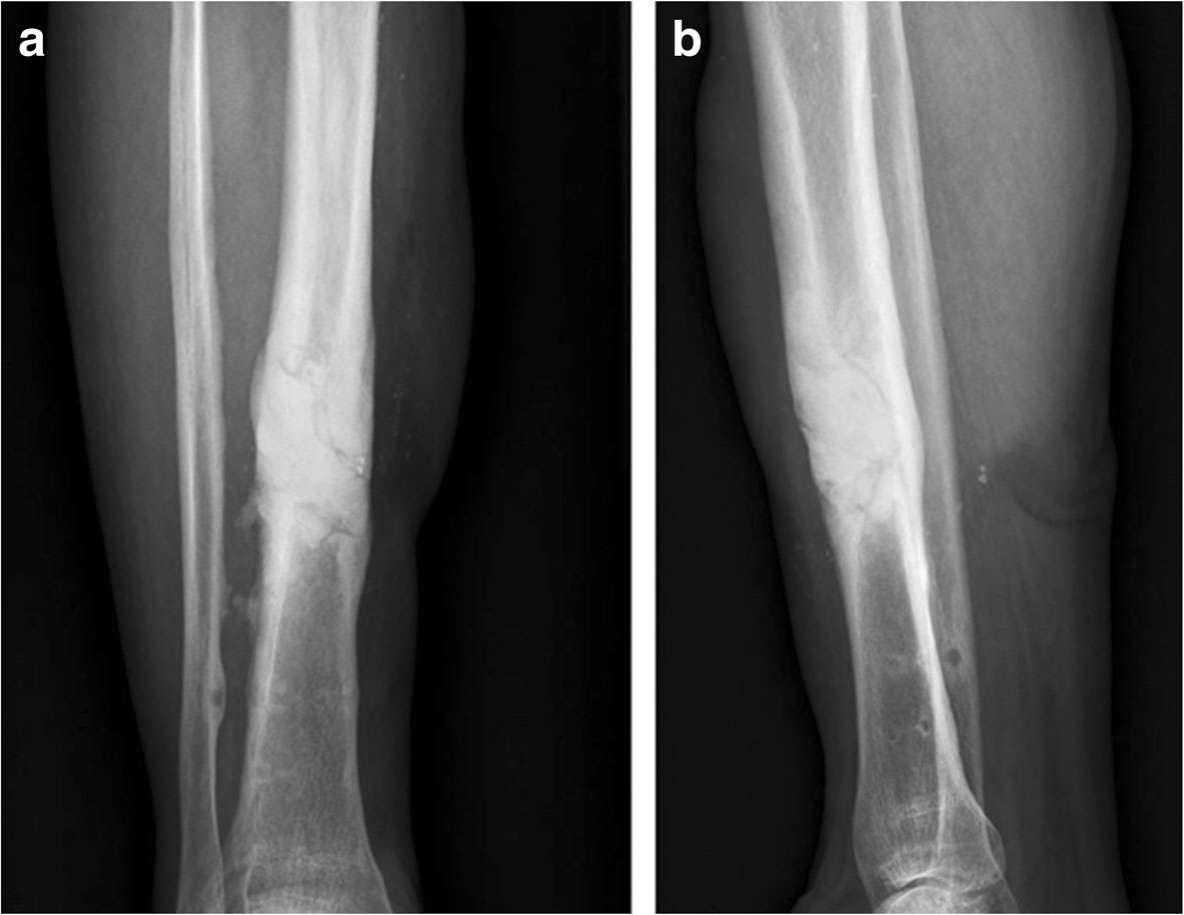
Postoperative radiographs showed that PMMA spacer was absorbed filled in the bone cavity after surgical treatment. (**a**) posteroanterior, (**b**) lateral

For the group treated with combination therapy vancomycin-loaded calcium sulfate pellets and vancomycin-loaded PMMA spacers, the vancomycin-loaded calcium sulfate pellets were implanted with monolayer morphology in the bony space after removal of the internal fixation material and thorough surgical debridement. The vancomycin-loaded PMMA spacers before hardening were subsequently filled in the cavity. At last, the vancomycin-loaded calcium sulfate pellets were implanted in the other side of the PMMA spacers’s surface without calcium sulfate pellets embedded. The above procedure ensured that the calcium sulfate pellets were embedded in the surface of the PMMA spacers and cannot be completely embedded in the PMMA cement, which would facilitate the degradation of calcium sulfate pellets and the release of antibiotics loaded in calcium sulfate pellets (Fig. 1). In the other group, the vancomycin-loaded PMMA spacers before hardening were implanted in the bony space after removal of the internal fixation material and thorough surgical debridement (Fig. 2).

### Systemic antibiotic therapy

Preoperative or postoperative bacterial culture and bacterial classification were performed in all patients (Table 1). Preoperatively, all patients of the two groups received combinations of two kinds of antibiotics for 1week in order to achieve better control of infection, whether or not the result of the bacterial culture was informed. Preoperative antibiotic coverage comprised of Cefazolin (24 cases), Ceftazidime (10 cases), Ciprofloxacin (18 cases), Levofloxacin (16 cases), Clindamycin (13 cases), Amikacin (15 cases), Oxazocilline (13 cases). Postoperatively, two kinds of intravenous antibiotics were chosen based on the results of the bacterial culture of tissues obtained during surgical debridement and administered for 2 to 3 weeks. ostoperative antibiotic coverage included Cefazolin (18 cases), Cefatriaxone (9 cases), Ceftazidime (6 cases), Ciprofloxacin (9 cases), Levofloxacin (10 cases), Clindamycin (9 cases), Amikacin (8 cases), Oxazocilline (6 cases), Fosfomycin (5 cases), Vancomycin (5 cases), Rifampicin (8 cases). Oral antibiotic therapy was not administrated in all patients.

**Table 1:** Microbiology of the two groups before surgery and after surgery

Types of bacteria	The combination therapy group (*n* = 26)		The PMMA group (*n* = 25)	
	preoperation	postoperation	preoperation	postoperation
Staphylococcus aureus	10	1	9	2
Staphylococcus warneri	1	-	2	-
Pseudomonas aeruginosa	3	1	3	2
Escherichia coli	3	1	2	1
Proteus mirabilis	2	-	2	-
Serratia marcescens	-	-	1	-
Enterobacter cloacae	-	-	1	-
klebsiella pneumoniae	3	-	1	-
MRSA	1	1	1	1
Total	23	4	22	6

*P* > 0.05

### Result evaluation

The safety and effectiveness of combination therapy with vancomycin-loaded calcium sulfate pellets and vancomycin-loaded PMMA spacers, compared with vancomycin-loaded PMMA spacers after the first-stage surgical treatment, were evaluated mainly through serologic assessment, eradication of infection, recurrence rate of infection and reoperation rate because of infected persistence or infected recurrence. The rate of complications and adverse events in the combination therapy group, comparing with the PMMA spacers group, was employed to evaluate safety. Absence of infection after the first-stage surgical treatment was defined as the clinical healing of incision without local drainage or erythema/swelling/pain, no systemic fever, and lacking of evidence of infection on serologic tests (elevated leukocyte, erythrocyte sedimentation rate and C-reactive protein) or imaging examination (evidence of progressive destruction of the bone). The PMMA spacers had to be removed approximately 2 months after complete absorption of calcium sulfate pellets and absence of infection resulted from the first-stage surgical treatment, and the second-stage surgical revision were subsequently performed. The effectiveness after the second-stage surgical revision were assessed mainly through recurrence rate of infection and the healing and growing of the bone graft implanted in the bone defect area.

### Follow-Up

The average follow-up after the first-stage surgery was 24 months (range, 15–48 months). Bacterial, serologic, clinical and radiologic evaluation were performed immediately and at 3 days, 1 weeks, 2 weeks, 1 month, 2 months, 3 months, 6 months, 1 year, and 2 years after the first-stage surgical treatment.

### Statistical analysis

The SPSS (Version 16) software package (SPSS Inc, USA) was employed for statistical analysis. Continuous variables such as age and leucocyte count were statistically analyzed using Student *t* test. Categorical variables such as sex and absence or presence of infection were statistically analyzed through Chi square test. If the expected variables were less than five, Fisher’s exact test was employed for statistical analysis. The level of statistical difference was defined at a *P* < 0.05 level.

## Results

### PMMA spacers group

#### Eradication of infection, rate of infection recurrence and reoperation rate

For those patients treated with vancomycin-loaded PMMA spacers, absence of infection was achieved in 16 of 25 (64.00%) patients at 2 months after the first-stage surgical treatment, according to the definition of the study. Persistent infection occurred in four patients and recurrence of infection occurred in five patients after the first-stage surgical therapy. The reoperation rate because of persistent or recurrent infection after the first-stage surgery was 36.00% (9 of 25).

#### Additional surgical therapy related to persistent or recurrent infection

Four cases required additional surgical therapy for a total of 12 additional procedures because of persistent infection after the first-stage surgical treatment. One of the four cases achieved eradication of infection. Five cases underwent additional surgical therapy for a total of 14 additional procedures because of recurrent infection after the first-stage surgical treatment. The recurrence of infection occurred more than 3 months postoperatively. One of the five cases achieved eradication of infection. In the additional surgical treatment, multiple consecutive operative steps were performed. These steps included removing the previously implanted PMMA spacers, debridement of infected tissue, and elimination of dead space with vancomycin-loaded PMMA spacers again. Two patients required amputation due to persistent or recurrent infection in this group.

#### Combination therapy with calcium sulfate pellets and PMMA spacers group eradication of infection, rate of infection recurrence and reoperation rate

For those patients treated with combination therapy with vancomycin-loaded calcium pellets and vancomycin-loaded PMMA spacers, infection was primarily eradicated in 92.31% of patients (24 of 26 cases) based on the definition of this study after the first-stage surgical treatment. Two patients underwent further surgical operation because of recurrent infection in the follow-up. One of the two cases achieved eradication of infection, with a secondary infection eradication rate of 96.15% (25 of 26). The reoperation rate because of recurrent infection was 7.69% (2 of 26) after the first-stage surgery. There were no persistent infections in this group.

#### Additional surgical therapy related to persistent or recurrent infection

Two cases required additional surgical therapy for a total of 4 additional procedures because of recurrent infection after the first-stage surgical treatment. One of the two cases achieved eradication of infection. The recurrence of infection occurred more than 3 months postoperatively. One of the two cases achieved eradication of infection. In the additional surgical treatment, multiple consecutive operative steps were performed. These steps included removing the previously implanted combination with calcium sulfate pellets and PMMA spacers, debridement of infected tissue, and elimination of dead space with combination with calcium sulfate pellets and vancomycin-loaded PMMA spacers again. One patients required amputation due to recurrent infection in this group.

#### Calcium sulfate pellet resorption

**Fig. 3:**
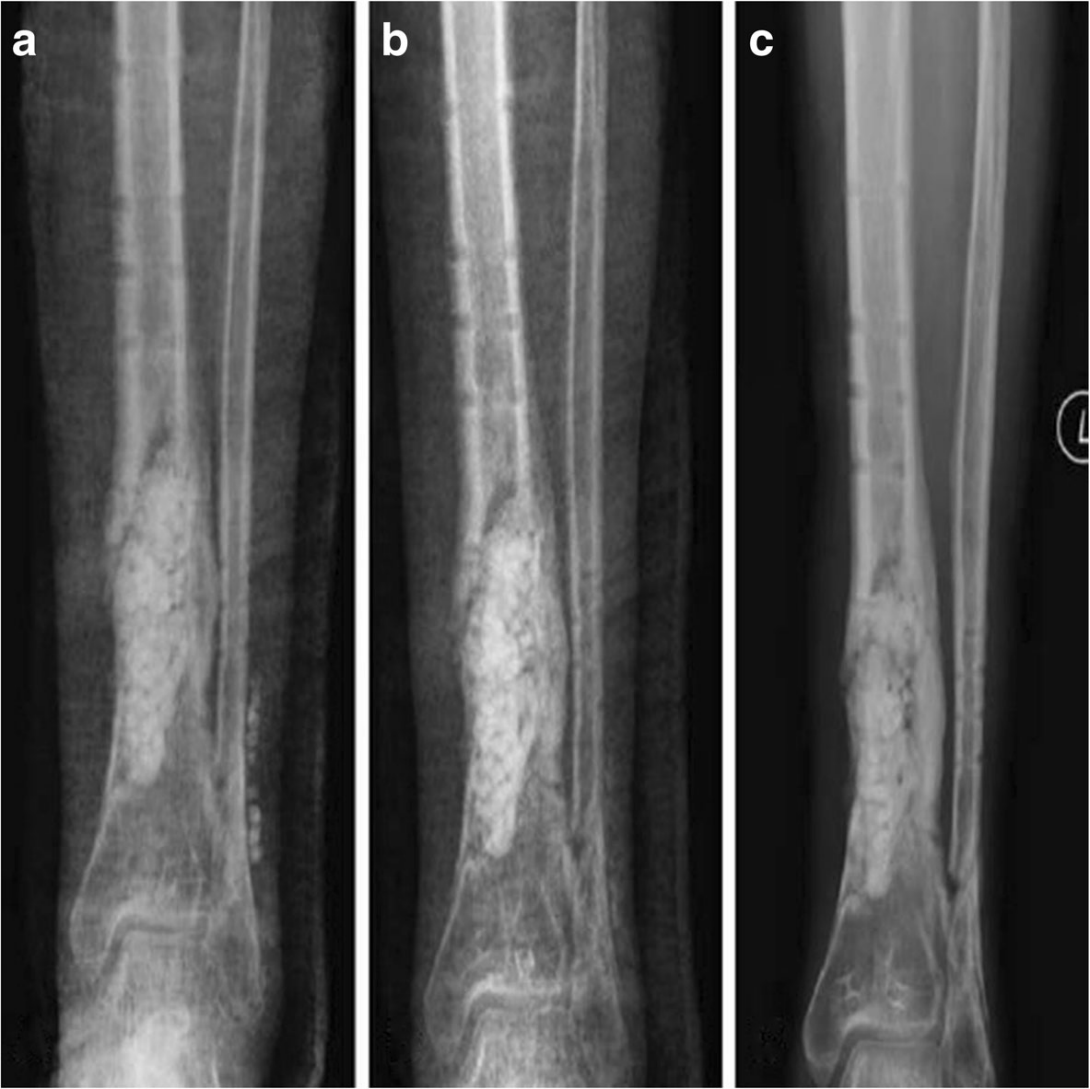
**a**-**c** Postoperative radiographs showed that the combination of calcium sulfate pellets and PMMA spacer was filled in the bone cavity after surgical treatment, and the calcium sulfate pellets were absorbed at an average of 6 weeks (range, 30–60 days)

The calcium sulfate pellets were completely absorbed at an average of 6 weeks (range, 30–60 days) in all patients (Fig. 3). The calcium sulfate pellets were radiographically invisible at 4 weeks (three case), 5 weeks (five cases), 6 weeks (six cases), 8 weeks (five cases).

#### Hematological parameters and histopathological inspection in the two groups

In the two groups, the median leukocyte counts, value of C- reactive protein (CRP), and erythrocyte sedimentation rate (ESR) were abnormally increased within 1 week after the first-stage surgical treatment, and then gradually returned to normal at 2 months postoperatively, except for the persistent or recurrent infection, laboratory examination showed no nephrotoxic or hepatotoxic effects in each group. All the patients in the two groups were diagnosed of chronic osteomyelitis through histopathological inspection postoperatively.

#### Complications of the two groups after surgery

The complication in the vancomycin-loaded PMMA spacers group after surgery included persistent drainage from the wound, nail infection of external fixation, thromboembolism and recurrent infection. Four patients had the complication of persistent drainage from the wound after the first-stage surgery. Two patients had the complication of recurrent infection, two patients had the complication of nail infection of external fixation. And one patient had the complication of thromboembolism in the combination therapy with vancomycin-loaded calcium sulfate pellets and vancomycin-loaded PMMA spacers group. None of all the patients in the two groups had the complications of seroma formation, nephrotoxic effects, Hepatotoxic effects, Gastrointestinal side effects, etc. (Table 2). There was a statistic difference in complications after the first-stage surgery between the two groups.

**Table 2:** postoperative complications of the two groups

complications	The combination therapy group (*n* = 26)	The PMMA group (*n* = 25)
Persistent drainage from the wound	0	4
Secondary infection	0	2
Nail infection of external fixation	0	2
Fractures	0	0
Seroma formation	0	0
Nephrotoxic effects	0	0
Hepatotoxic effects	0	0
Neuropraxia	0	0
Gastrointestinal side effects	0	0
Thromboembolism	1	0

*P* < 0.05

### Statistical analysis

There was no statistic difference about all the variables in the two groups comparing them with each other (Table 3). After the first-stage surgery, the number of patients with infection eradication in the combination therapy with vancomycin-loaded calcium sulfate pellets and vancomycin-loaded PMMA spacers group was more than the vancomycin-loaded PMMA spacers group (combination therapy group, 24 versus PMMA group, 16; *P* = 0.034) (Table 4), and the number of patients requiring repeat operation due to persistent or recurrent infection in the combination therapy with vancomycin-loaded calcium sulfate and vancomycin-loaded PMMA group was less than the vancomycin-loaded PMMA group (combination therapy group,2 versus PMMA group, 9; *P* = 0.034). After repeat operation due to persistent or recurrent infection that happened after the first-stage surgery, the total number of patients with infection eradication in the combination therapy with vancomycin-loaded calcium sulfate pellets and vancomycin-load PMMA spacers group was more than the vancomycin-loaded PMMA spacers group (combination therapy group, 25 versus PMMA group, 18; *P* = 0.047) (Table 5). For the local infection recurrence groups, there was no statistic difference in the two groups (Table 6)

**Table 3:** Comparison of the Variables in the Two Groups

Variables		The combination therapy Group (*n* = 26)	The PMMA Group (*n* = 25)
gender	Male	14	14
	Female	12	11
Mean age (years)		43.84 (range, 15–64)	42.32 (range, 17–68)
Categories	Chronic post-traumatic osteomyelitis	14	13
	Chronic postoperative osteomyelitis	12	12
Location of void/defect	Femur	4	5
	Tibia	19	17
	Calcaneus	3	3
Volume of bone defect after debridement		62.73 (range, 10.3–163)	63.11 (range, 11.7–168)
Cierny-Mader classification	Stage1—medullary osteomyelitis	11	10
	Stage 2—superficial osteomyelitis	0	0
	Stage 3—localized osteomyelitis	9	8
	Stage 4—diffuse osteomyelitis	6	7
Infected nonunion		10	10
Number of external fixation		18	17

**Table 4:** The Number of Patients with Infection Eradication after the First-stage Surgery

Group	Infection Eradication	Persistent or Recurrent Infection	Total	Percentage (%)
PMMA Group	16	9	25	64
Combination Therapy Group	24	2	26	92.31
Total	40	11	51	78.43

Chi square test, *P* = 0.034

**Table 5:** The Total Number of Patients with Infection Eradication after Repeat Operation

Group	Infection Eradication	Persistent or Recurrent Infection	Total	Percentage (%)
PMMA Group	18	7	25	72
Combination Therapy Group	25	1	26	96.15
Total	43	8	51	84.31

*P* = 0.047

**Table 6:** Information of the local infection recurrence in the two group

		The combination therapy group	The PMMA group
Number of infection recurrence		2	5
Femur		0	1
Tibia		2	4
Cierny-Mader classification	Stage 1—medullary osteomyelitis	1	2
	Stage 3—localized osteomyelitis	1	3
Chronic post-traumatic osteomyelitis		1	3
Chronic postoperative osteomyelitis		1	2
bacterial type	S.aureus	1	3
	MRSA	0	1
	Pseudomonas aeruginosa	1	1
	Infected nonunion	2	4
	Number of external fixation	2	4
Antibiotic given	Levofloxacin	1	1
	Cefazolin	0	3
	Ceftazidime	1	1

*P* > 0.05

## Discussion

The significant progress has been made in orthopedic surgery and antibiotic therapy over the past few decades, however, the treatment of chronic post-traumatic and postoperative osteomyelitis is still a difficultly controllable clinical problem. It is considerably difficult to treat because of the factors of patient and pathogen. Besides radical surgical debridement, appropriate treatments consist of long-term systemic antibiotic therapy, local antibiotic therapy by local delivery devices which are composed of biodegradable or non-biodegradable materials, and sometimes removal of internal fixation. In the study, in order to reduce serious side effects and the occurrence of bacterial resistance, intravenous antibiotic therapy was administrated for only 3 to 4 weeks. Preoperatively, it was used for 1 week, while it was used for 2 to 3 weeks postoperatively. Nephrotoxic effects, hepatotoxic effects and other adverse reactions related to intravenous antibiotic therapy did not appear in this study. Generally, systemic antibiotic treatment cannot achieve sufficient release of antibiotic in the area of infected bone due to poor blood supply resulting from soft tissue scarring and bone sclerosis.

Compared with systemic antibiotic treatment, local antibiotic delivery systems implanted with non-biodegradable or biodegradable delivery devices possess several merits, because effective and higher antibiotic concentrations are obtained in the local area of infected bone through the systems for a prolonged period of time. In addition, the period of patient hospitalisation is significantly shortened, the adverse events related to systemic chemotherapy are availably prevented, and the risk of systemic toxicity can be reduced [14]. Local antibiotic therapies play a critical role in the treatment of chronic osteomyelitis. So, local application of bone void filler implanted with antibiotic indicated adequate local concentration of the antibiotic in the area of infected bone, simultaneously, sufficient low systemic exposure in patients suffering from chronic osteomyelitis. It has been revealed, after local application of antibiotic delivery devices, effective antibiotic concentration in the area of infected bone is available for up to 6 weeks in the surrounding tissue.

There are two types of antibiotic delivery devices, non-biodegradable delivery system and biodegradable delivery system. Frequently, the non-degradable antibiotic delivery system is consisted of PMMA carriers. Higher local antibiotic concentrations can obtain by the temporary usage of the antibiotic delivery devices, while lower systemic concentration of antibiotic can achieve, minimizing adverse events. Therefore, PMMA has rapidly turned into a standard protocol for local antibiotic delivery in the treatment of osteomyelitis. PMMA loaded with antibiotic are often used in the form of spacers, which not only increased the local concentration of antibiotics in the infected area, simultaneously minimized the level of systemic antibiotics, but also eliminated the void cavity caused by surgical debridement [14]. Furthermore, it provided immediately structural stabilization [15] that may be advantageous to the treatment of osteomyelitis. PMMA loaded with antibiotic is established as a standard in the treatment of chronic osteomyelitis, which can offer higher local antibiotic concentrations, but it has its own disadvantages, mainly because it shows burst release and consequently sub-therapeutic release kinetics, especially the level of eluted antibiotic declines over time [16]. Therefore, to achieve and maintain the desired level of the antibiotic is very difficult at the expected length of time. When the release levels of antibiotic loaded in PMMA are less than the minimum inhibitory concentration, PMMA carriers themselves can represents a potential nidus for infection and offer a substratum for bacterial colonization and biofilm formation. Bacteria in biofilms are less sensitive to antibiotics, because bacterial biofilms can impede the penetration of antibiotics [17], which may promote progression and recurrence of chronic infection. Comparing with their planktonic counterparts, bacteria in biofilms presented a 1,000-fold tolerance to antibiotics, and significant resistance to host immunity [18]. This may be a part of the reason why a growing number of osteomyelitis relapses occur and antibiotic resistance is becoming increasingly prevalent. Bacterial adhesion and sustained growth of bacteria on the surface of the antibiotic-loaded PMMA have been deeply studied [19–21].

The research for biodegradable alternatives have become highly necessary due to these drawbacks. It is a strongly attractive alternative that the application of biodegradable devices for antibiotic delivery in the treatment of chronic osteomyelitis. Degradable devices produce a zero-order release kinetics, which will not promote the growth of antibiotic-resistant bacterial strains because of lack of long-term and sub-therapeutic concentration tail-release. A reviews including 15 studies showed an outcome range from 80 to 100% eradication in all patients where infection was absent after treatment with the use of antibiotic - impregnated bioabsorbable bone substitutes [22]. Calcium sulfate is one kind of biodegradable delivery devices which can be loaded with antibiotics. The advantages of calcium sulfate is that it can offer the chance to delivery an effective concentration of local antibiotics with the biodegradation after implantation [23–26]. In contrast to PMMA spacers, the porous structure of calcium sulfate can make the antibiotic to sufficiently penetrate, it can be absolutely absorbed in the human tissue and has more adequate elution of antibiotics, moreover, it does not promote inflammation [27–30]. Calcium sulfate can completely release antibiotic load in them after degradation, while absence of substratum for bacterial colonization. Recently, one study revealed that antibiotic-loaded calcium sulfate beads were able to prevent bacterial colonization, and effectively reduce biofilm formation [31]. However, calcium sulfate has its own inherent shortcomings. The absorption of calcium sulfate can occur rapidly in vivo, and the mechanical strength of the material can quickly lose with degradation [32, 33]. So, calcium sulfate are not intended to provide structural support. The degradation products of calcium sulfate carriers generally resulted in persistent drainage from the wound which may aggravate deep infection [34]. Calcium sulfate absorbs plenty of water, subsequently promote seromas formation and increase the risk of secondary infection [35]. Additionally, calcium sulfate pellets cannot be expected to replace PMMA spacers in situations where mechanical support and integrity are important to the procedure (such as spacers in staged revision).

it is envisioned that vancomycin-loaded calcium sulfate pellets can be employed in combination with vancomycin-loaded PMMA spacers in the treatment of osteomyelitis, with calcium sulfate pellets providing the opportunity to deliver higher local antibiotics concentrations with degradation and PMMA spacers providing structural strength and integrity. Radiographic analysis showed that the calcium sulfate pellets were resorbed completely in approximately 30–60 days in this study. In a recent study, local implantation of calcium sulfate beads loaded with antibiotic obtained a good therapeutic effect in the treatment of the lower-extremity osteomyelitis in the absence of systemic antibiotics [35]. In this study, we applied the advantages of PMMA spacers and calcium sulfate pellets respectively to yield synergistic anti-infection effect. The combination therapy with vancomycin-loaded calcium sulfate pellets and vancomycin-loaded PMMA spacers presented a good effect in the treatment of chronic post-traumatic and postoperative osteomyelitis in this study, comparing with PMMA spacers loaded antibiotic. In this study, the group of the combination therapy with calcium sulfate pellets and PMMA spacers showed less rate of persistent or recurrent infection and rate of reoperation because of persistent or recurrent infection than the group of PMMA spacers after the first-stage treatment (*P* < 0.05).

The PMMA spacers loaded with a combination of different antibiotics would potentially increase the antimicrobial spectrum. It has been revealed that PMMA cement loaded with more than one antibiotic could elevate antibiotic elution, compared to only containing one antibiotic [36, 37]. So, in the study vancomycin was loaded in PMMA power with the addition of gentamicin sulphate in order to produce a synergistic effect. In a recent paper, calcium sulfate beads loaded with more than one antibiotic could elevated and prolonged elution of vancomycin, compared with beads only containing vancomycin [38].

### Weakness of this study

This study itself had several limitations consisted of a non-randomized trial, small samples and absence of measurement of the local levels of antibiotic. Thus, a large-sample randomized controlled clinical trial is very necessary to evaluate the effect of the combination therapy with calcium sulfate pellets loaded antibiotic and PMMA spacers loaded antibiotic in the treatment of chronic post-traumatic and postoperative osteomyelitis.

## Conclusions

In conclusion, the study demonstrated the feasibility and efficacy of the combination therapy with calcium sulfate pellets loaded antibiotic and PMMA spacers loaded antibiotic in treatment of chronic post-traumatic and postoperative osteomyelitis.

## Abbreviations

PMMA: Polymethyl methacrylate
